# Global mitochondrial protein import proteomics reveal distinct regulation by translation and translocation machinery

**DOI:** 10.1016/j.molcel.2021.11.004

**Published:** 2022-01-20

**Authors:** Jasmin Adriana Schäfer, Süleyman Bozkurt, Jonas Benjamin Michaelis, Kevin Klann, Christian Münch

**Affiliations:** 1 Institute of Biochemistry II, Goethe University Frankfurt, Theodor-Stern-Kai 7, Haus 75, 60590 Frankfurt am Main, Germany; 2 Frankfurt Cancer Institute, Frankfurt am Main, Germany; 3 Cardio-Pulmonary Institute, Frankfurt am Main, Germany

**Keywords:** proteomics, SILAC, TMT, mitochondria, protein translocation, disease, integrated stress response, respiratory chain complexes, translation, proteostasis

## Abstract

Most mitochondrial proteins are translated in the cytosol and imported into mitochondria. Mutations in the mitochondrial protein import machinery cause human pathologies. However, a lack of suitable tools to measure protein uptake across the mitochondrial proteome has prevented the identification of specific proteins affected by import perturbation. Here, we introduce mePROD^mt^, a pulsed-SILAC based proteomics approach that includes a booster signal to increase the sensitivity for mitochondrial proteins selectively, enabling global dynamic analysis of endogenous mitochondrial protein uptake in cells. We applied mePROD^mt^ to determine protein uptake kinetics and examined how inhibitors of mitochondrial import machineries affect protein uptake. Monitoring changes in translation and uptake upon mitochondrial membrane depolarization revealed that protein uptake was extensively modulated by the import and translation machineries via activation of the integrated stress response. Strikingly, uptake changes were not uniform, with subsets of proteins being unaffected or decreased due to changes in translation or import capacity.

## Highlights

• Proteomics approach to quantify protein uptake into mitochondria
• Determination of protein uptake rates for >700 mitochondrial proteins
• Characterization of differential protein uptake changes during mitochondrial stress
• Protein translation and translocation integrate to change protein uptake

## Graphical abstract

**Figure:**
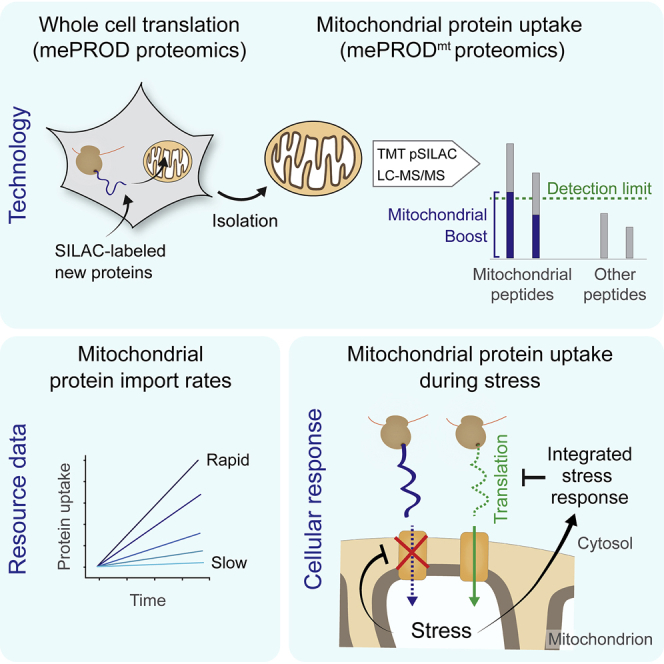

## Teaser

Schäfer et al. describe a proteomics method to quantify mitochondrial protein uptake for >700 mitochondrial proteins. They characterize how protein import inhibitors affect protein uptake. This revealed that changes in protein translation and translocation integrate to control mitochondrial uptake for different sets of proteins and submitochondrial compartments.

## Introduction

Mitochondria serve essential cellular functions, including energy production and anabolic pathways. Due to their endosymbiotic origin, mitochondria possess their own genome; however, autonomy was lost upon the relocation of the majority of genes encoding for the >1,000 mitochondrial proteins to the nucleus (Brandvain and Wade, 2009; Rath et al., 2021). As a consequence, nuclear-encoded mitochondrial proteins are translated in the cytosol and therefore require subsequent mitochondrial import. Thus, the rate of mitochondrial protein uptake into mitochondria is largely defined by translation rates and capacity of the import machinery.

Mitochondria consist of two membranes that define four distinct suborganellar compartments, which all accommodate proteins: the outer mitochondrial membrane (OMM), the intermembrane space (IMS), the inner mitochondrial membrane (IMM), and the mitochondrial matrix. Multiple protein import machineries evolved that specialized on protein trafficking to these mitochondrial compartments (Liu et al., 2011). Numerous studies led to a detailed understanding of their composition and how these machineries mechanistically mediate protein translocation. However, a comprehensive characterization of mitochondrial import of the pool of nuclear-encoded mitochondrial proteins remains missing. This information will be crucial to understand the effects of mutations in import machinery components that are associated with various diseases (Kang et al., 2018; Nicolas et al., 2019).

Conditions of mitochondrial dysfunction have been shown to induce the integrated stress response (ISR). ISR activation results in transcriptome changes and attenuation of cytosolic translation. While the ISR-mediated transcriptome and translatome changes have been well studied (Quirós et al., 2017; Klann et al., 2020), the resulting effects on mitochondrial protein import, and thus the mitochondrial proteome, remain unknown. A key limitation for analyzing the effects of disease mutations or ISR activation on reshaping mitochondrial uptake across the mitochondrial proteome is that mitochondrial protein import measurements are largely limited to the analysis of single proteins (e.g., mitochondrially targeted GFP, radiolabeled proteins *in vitro*) (Yano et al., 2014; Di Maio et al., 2016; Liu et al., 2018), which does not allow monitoring import rates across the whole mitochondrial proteome. The development of unbiased import assays covering the whole proteome in live cells is crucial to determine specific effects on protein subpopulations and to define differences across disease conditions or stresses.

To overcome these limitations, here, we describe an assay based on pulsed-stable isotope labeling by amino acids in cell culture (SILAC) quantitative proteomics for globally measuring mitochondrial uptake of newly synthesized proteins. For this assay, we adapted multiplexed enhanced protein dynamics (mePROD), which was recently established for acute translation measurements (Klann et al., 2020). The mePROD method combines pulsed-SILAC labeling with tandem-mass tag (TMT)-based multiplexing, including a fully SILAC-labeled booster channel to increase the sensitivity for heavy labeled peptides. Here, we show that compartment-specific boost signals allowed us to specifically enhance signals for mitochondrial protein translation and import. Applying this method to cells treated with inhibitors of different mitochondrial import mechanisms revealed specific subsets of mitochondrial proteins dependent on these pathways. Strikingly, we found that mitochondrial depolarization caused differential effects on three groups of proteins. The first group showed decreased uptake directly due to a loss of import capacity. The second group contained proteins for which reduced uptake was driven by a reduction in translation resulting from ISR activation. The third group of proteins was not affected by the loss of membrane potential. Using an unbiased approach to profile the dynamics of protein uptake sheds new light on how protein import and translation cooperate to regulate mitochondrial function. To ease the broad use of the method, we additionally provide a standalone browser application and a Python package for easy data analysis.

## Results

### Compartment-specific signal boosting of cell-wide and organelle-selective pulsed-SILAC experiments

**Figure 1:**
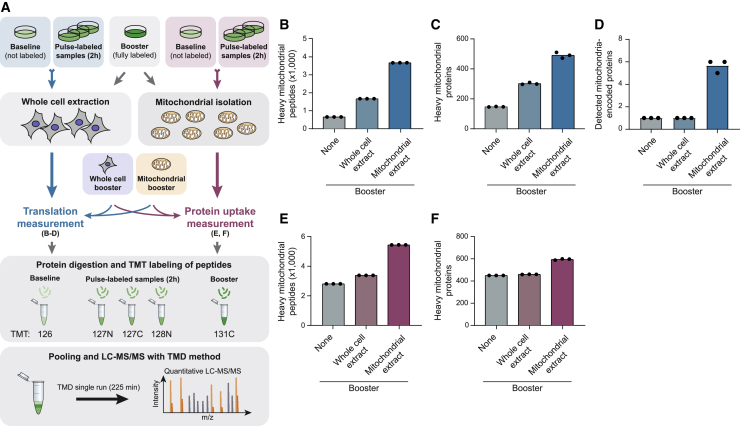
Compartment-specific signal boosting of cell-wide and organelle-selective pulsed-SILAC experiments (A) Workflow of cell-wide (left, blue) and mitochondria-selective (right, magenta) pulsed-SILAC proteomics for measuring translation or uptake of mitochondrial proteins, respectively. A non-labeled baseline sample and samples of cells pulse labeled for 2 h were subjected to whole-cell extraction (left) for translation measurement or mitochondrial isolation (right) for measuring protein uptake. Equally, cells of a fully SILAC-labeled booster sample were subjected to whole-cell extraction or mitochondrial isolation to yield a whole-cell or mitochondrial booster. Baseline and pulse-labeled samples for translation or protein uptake measurements were complemented with a whole-cell or mitochondrial booster to improve the sensitivity for all or mitochondrial proteins. Proteins were digested, labeled with tandem mass tag (TMT)11, pooled, and measured by LC-MS/MS with targeted mass difference (TMD). (B–D) Shown are numbers of heavy SILAC-labeled mitochondrial peptides (B), proteins (C), or mitochondrially encoded proteins (D) dependent on the addition of no booster or booster signals derived from whole-cell or mitochondrial extracts (n = 3 in 1 multiplex). (E and F) Number of heavy SILAC-labeled mitochondrial peptides (E) or proteins (F) from purified mitochondria identified in combination with no booster or booster signals derived from whole-cell or mitochondrial extracts (n = 3 in 1 multiplex). See also Figure S1.

Our previous study established mePROD as a method to boost the signal of all heavy (i.e., newly synthesized) peptides in a cell lysate to monitor global translation (Klann et al., 2020). We hypothesized that this approach should also allow boosting the signal of specific subsets of heavy peptides, such as all peptides originating from one compartment, within a cell lysate. To test this hypothesis, we performed mePROD experiments to measure the translation of proteins from whole-cell extracts (WCEs) with or without addition of a booster channel derived from WCE or purified mitochondria from fully SILAC-labeled HeLa cells (Figure 1A). Addition of a mitochondrial booster signal doubled the number of mitochondrial peptides for which heavy peptides could be quantified, indicating that the addition of a selective booster signal enables measuring translation of specific parts of the proteome (Figures 1B and 1C). Strikingly, this approach also allowed us to measure translation of 6 of the 13 mitochondrially encoded proteins within WCE by a single liquid chromatography-tandem mass spectrometry (LC-MS/MS) run (Figure 1D).

We next asked whether the mePROD approach could be modified to measure protein uptake into mitochondria. Mitochondrial proteins are translated in the cytosol and rapidly imported (Avendaño-Monsalve et al., 2020). Thus, we reasoned that treating cells with a SILAC pulse, followed by the isolation of mitochondria and measurement of labeled peptide abundance inside mitochondria would give a direct and global readout of mitochondrial protein uptake. To test this approach, we SILAC pulse labeled cells and carried out mitochondrial isolation before pooling samples into a mePROD setup and analysis by a single LC-MS/MS run using targeted mass difference (TMD) (Klann and Münch, 2020) (Figure 1A). Mitochondrial proteins were highly enriched upon mitochondrial isolation, while cytosolic proteins were largely absent (Figures S1A and S1B). Proteinase K treatment only slightly affected co-isolation of other organelles; however, it led to a loss of OMM proteins (Figures S1C and S1D). Therefore, we did not include proteinase K in the standard procedure and filtered for mitochondrial proteins using MitoCarta3.0 instead (Rath et al., 2021). In this setup, the addition of the mitochondrial booster signal nearly doubled the amount of mitochondrial heavy peptides quantified to yield ∼600 quantified mitochondrial proteins (Figures 1E and 1F) and established that the mitochondrial proteome-targeting mePROD method (mePROD^mt^) may serve as a proteomic mitochondrial uptake assay.

### Characterization of protein uptake kinetics across the mitochondrial proteome

**Figure 2:**
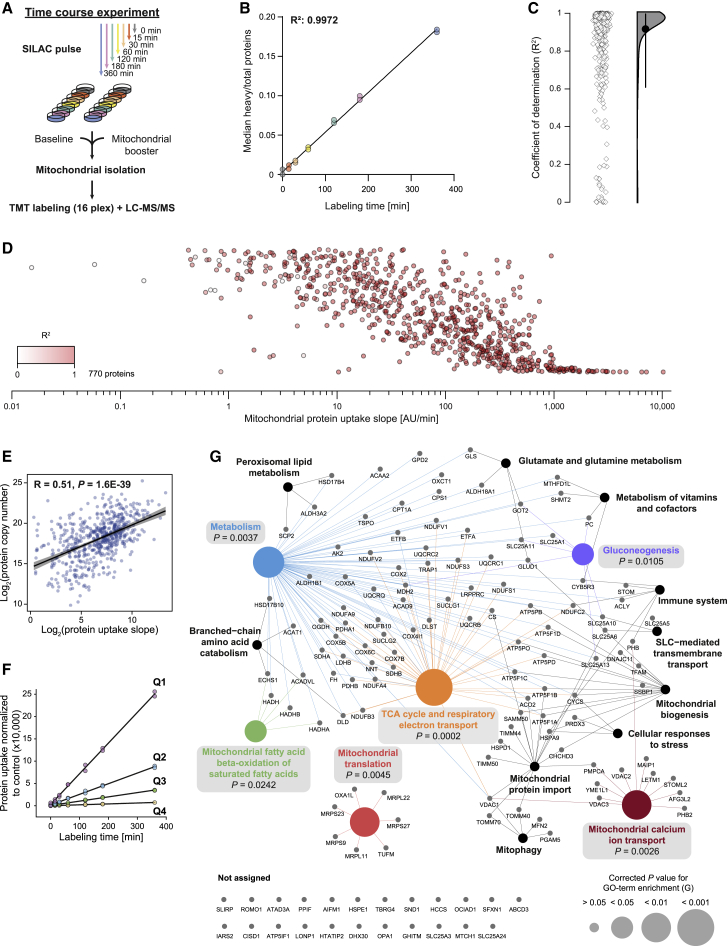
Characterization of protein uptake kinetics across the mitochondrial proteome (A) Experimental scheme of a time course experiment to determine mitochondrial protein uptake kinetics. Cells were pulsed-SILAC-labeled for 0–360 min in duplicate and then subjected to mitochondrial isolation, TMT labeling, and measurement by LC-MS/MS with targeted mass difference selection. (B) Plot of heavy-to-total protein ratios of proteins over time (n = 2). (C) Coefficients of determination (R^2^) of the uptake slope determined for each identified mitochondrial protein, shown as individual data points and half-violin plot. (D) Plot of individual uptake slopes of all identified mitochondrial proteins. Color of data points represents R^2^ value of the respective uptake slope. Data points are scattered along the vertical axis to prevent excessive overlapping. a.u., arbitrary unit. (E) Pearson correlation of protein uptake slopes [log_2_] and protein copy numbers [log_2_]. R, Pearson coefficient. (F) Normalized protein uptake over time of mitochondrial proteins showing R^2^ ≥ 0.95 grouped into quartiles (Q1–Q4) based on their uptake slope (n = 2, see D). Data points show mean values of the quartiles over time. (G) Representation of Reactome pathway network for proteins within Q1 (from F). Network was obtained with the Cytoscape plug-in ClueGO. Pathways highlighted with colors and gray boxes were significantly enriched. p value corrected with Benjamini-Hochberg procedure. The full Gene Ontology (GO) term list is provided in Table S2. See also Figure S2 and Tables S1 and S2.

To assess the accuracy of the mePROD^mt^ protein uptake assay and examine the relative protein uptake rates across the mitochondrial proteome, we monitored uptake over time. Cells were SILAC pulse labeled for 15–360 min, mitochondria extracted, and samples analyzed in a mePROD^mt^ setup with a mitochondrial booster channel (Figure 2A). Mitochondrial heavy peptides displayed a linear increase over time and measurements were highly reproducible across replicates, demonstrating the high accuracy and robustness of the method (Figure 2B). Throughout the time course, median labeling values stayed below 20%, indicating that mitochondrial half-lives are longer than the monitored 360 min, consistent with previous half-life measurements (Schwanhäusser et al., 2011). Currently, comprehensive data of uptake speed and individual protein import behavior of the mitochondrial proteome are not available. Thus, we carried out linear least-squares regression modeling and extracted the slopes of the protein uptake kinetics and R^2^ values for quality control (Table S1). For the majority of proteins, curve fits were of high quality, represented by R^2^ values close to 1 (Figures 2C and S2A). When investigating proteins with lower correlation values, we identified some proteins, such as NDUFS6, TRUB2, CHCHD2, BNIP3, MRPS21, MCL1, and GADD45GIP1, that reached a plateau in protein uptake during the 6-h range (Figure S2A). This observation suggests that for these proteins, newly imported proteins did not replace old (i.e., light labeled) proteins anymore. Half-life calculations of these proteins confirmed a rapid turnover rate (Figure S2B), as had been shown for some of these proteins (Burstein et al., 2018; Chen et al., 2019; Szczepanowska and Trifunovic, 2021).

Strikingly, the dynamic range of protein uptake kinetics spanned six orders of magnitude (Figure 2D). To investigate whether these drastic differences arise due to biophysical protein properties, we correlated protein uptake slopes with protein length, hydrophobicity, charge, and copy number (Figures 2E and S2C). Protein copy numbers were the only feature that showed moderate correlation with uptake slopes. To investigate possible functional determinants of high import rates, we next grouped all of the proteins according to their uptake kinetics into 4 quantiles and examined the proteins contained in the top quartile (Q1; Figures 2F and 2G; Table S1). Reactome-based network analysis revealed that a substantial part of this group was involved in metabolic pathways (Figure 2G; Table S2). However, the same analysis of the quartile with the lowest import rates (Q4) showed a partial overlap of pathways, indicating that uptake rates of functionally related proteins were not strictly co-regulated (Figure S2D; Tables S1 and S2). In addition, assessment of key mitochondrial protein machineries showed no distinctive patterns (Figure S2E). However, some key components of the mitochondrial protein quality control system, including the chaperonins (HSPD1 and HSPE1) and mitochondrial HSP70 (HSPA9) were among the proteins showing the highest uptake rates (Figure S2E), highlighting the importance of constant protein influx for proteostasis.

We found that mePROD^mt^ was able to obtain highly reproducible quantification of mitochondrial uptake rates for hundreds of proteins, allowing kinetic analyses of mitochondrial protein uptake.

### Mitochondrial uptake proteomics reveal complex protein uptake patterns upon perturbation

**Figure 3:**
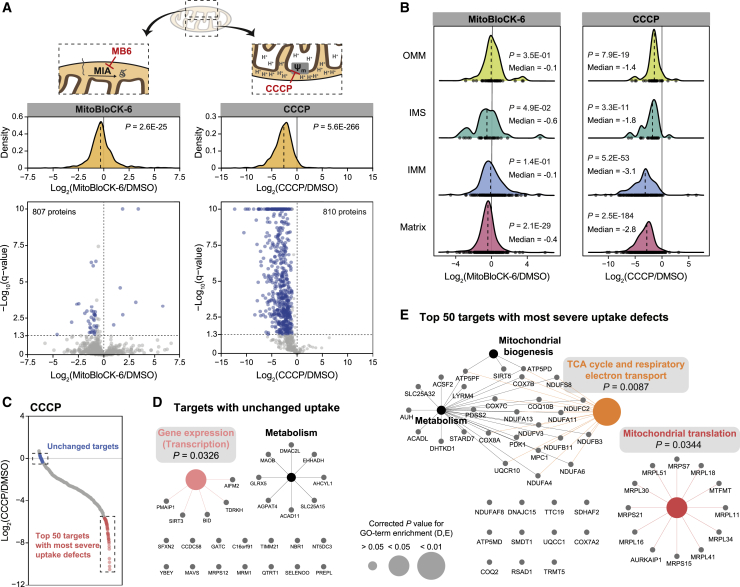
Mitochondrial uptake proteomics reveal complex protein uptake patterns upon perturbation (A) Illustration of the effects of MitoBloCK-6 (MB6) and CCCP on the uptake of proteins into the intermembrane space and matrix, respectively (top). Fold changes of mitochondrial protein uptake upon MitoBloCK-6 (left) or CCCP (right) treatment compared to DMSO-treated control cells, shown as density plots (upper) and volcano plots (lower) plotted against the adjusted p value (n = 3). Dashed lines indicate median values of the distributions. Data points of targets with significant changes (fold change [log_2_] ≤ −0.7 or ≥0.7, and adjusted p ≤ 0.05 for MitoBloCK-6; fold change [log_2_] ≤ −1 or ≥1, and adjusted p ≤ 0.05 for CCCP) are shown in blue. Adjusted p values > 10 were set to p = 10 for plotting; original adjusted p values are given in Tables S3 and S4. Kolmogorov-Smirnov test against normal distribution ∼0, with standard deviation obtained for the tested distribution. (B) Density plots of mitochondrial protein uptake fold changes upon MitoBloCK-6 (left) or CCCP (right) treatment compared to DMSO-treated control cells, based on suborganellar location (n = 3). Dashed lines indicate median values of the distributions. Dots at the bottom of the distributions indicate number and location of data points. OMM, outer mitochondrial membrane; IMS, mitochondrial intermembrane space; IMM, inner mitochondrial membrane; Kolmogorov-Smirnov test against normal distribution ∼0, with standard deviation obtained for the tested distribution. (C) Rank plot showing fold changes of mitochondrial protein uptake upon CCCP treatment compared to DMSO-treated control cells (n = 3). Unchanged targets (−0.35 ≤ fold change [log_2_] ≤ 0.35) and the top 50 targets showing the most severe uptake defects highlighted in blue and red, respectively. (D and E) Reactome pathway networks of targets with unchanged (−0.35 ≤ fold change [log_2_] ≤ 0.35) (D) or top 50 reduced (E) mitochondrial uptake upon CCCP treatment compared to DMSO-treated control cells. Networks were prepared with the Cytoscape plug-in ClueGO. Pathways highlighted with colors and gray boxes were significantly enriched. p value corrected with Benjamini-Hochberg procedure. Full GO term lists are provided in Table S2. See also Figure S3 and Tables S2, S3, and S4.

To further validate the mePROD^mt^ protein uptake assay, we applied it to examine protein uptake upon acute treatment with the import inhibitors MitoBloCK-6 and CCCP at commonly used, non-toxic concentrations (Figure S3A). MitoBloCK-6 selectively interferes with redox-regulated import of mitochondrial proteins by inhibiting the oxidase activity of GFER/Erv1 in the intermembrane space (Dabir et al., 2013). The protonophore CCCP disturbs mitochondrial homeostasis and broadly affects protein import by eliminating the inner mitochondrial membrane potential (Geissler et al., 2000) (Figure S3B). Global mitochondrial protein uptake was only slightly affected by treatment with MitoBloCK-6 for 6 h (Figure 3A, left; Table S3). However, analysis at the single-protein level revealed that 35 proteins were significantly reduced by up to 20-fold, whereas the uptake of 7 proteins was significantly increased. Several described targets of MitoBloCK-6 showed reduced uptake, two of which (CHCHD2 and CMC1) were significantly affected, indicating that MitoBloCK-6 was effective (Dabir et al., 2013; Modjtahedi et al., 2016) (Figure S3C). At levels not inducing cell death or pronounced mitophagy (Xiao et al., 2017), CCCP treatment for 2 h affected protein uptake extensively, causing a significant reduction in protein uptake globally and for 595 proteins at the single-protein level by up to 4,000-fold (Figure 3A, right; Table S4). We next evaluated these results by monitoring candidate proteins using western blotting. CCCP treatments longer than 2 h were required to be able to reliably detect and quantify the substiochiometric precursor proteins (Figure S3D). We observed reduced mitochondrial protein levels and accumulation of precursors in whole-cell lysates, consistent with our proteomics data (Figures S3E and S3F). To be able to specifically monitor newly synthesized mitochondrial proteins in cells, we fused candidate mitochondrial proteins with a HaloTag, which enables covalent labeling of the fusion protein in cells with differently modified HaloTag ligands. We expressed several Halo-fused mitochondrial proteins in cells and saturated all expressed HaloTag with an empty HaloTag ligand to then selectively label newly synthesized Halo-fusion proteins with biotinylated HaloTag ligand under control- or CCCP-treated conditions (Figure S3G). We followed mitochondrial protein uptake by comparing the ratio of pulse biotin-labeled, newly synthesized versus total protein levels of the fusion proteins during CCCP treatment. While less sensitive than mePROD^mt^, using this approach, we observed similar fold changes when compared to mePROD^mt^ (Figure S3H).

We next analyzed changes in mitochondrial protein uptake upon MitoBloCK-6 and CCCP treatment for each mitochondrial subcompartment individually to determine compartment-specific drug effects (Figure 3B). As expected, MitoBloCK-6 affected the uptake of IMS proteins (significant median uptake reduction of 34%) and additionally caused a milder effect on matrix proteins (Figure 3B). Treatment with CCCP caused a significant decline in protein uptake for all four mitochondrial subcompartments, with IMM and matrix proteins most severely affected showing median reductions of 90% and 85%, respectively (Figure 3B).

The strong effect of CCCP on overall mitochondrial protein uptake and the general importance of understanding the sets of proteins affected by membrane depolarization prompted us to analyze the impact of CCCP in more detail. Plotting uptake changes of mitochondrial proteins upon CCCP treatment against their uptake rates under steady-state conditions revealed no correlation between both parameters, as judged by Pearson correlation (Figure S3I). Hence, CCCP affected protein uptake in a way that went beyond a general and uniform reduction of uptake speed. The majority of all identified mitochondrial proteins showed a significant decrease in uptake; however, the uptake of 27 proteins remained nearly unchanged upon CCCP treatment (Figure 3C). Reactome pathway-based network analysis of these proteins revealed no specific pattern, with only the term gene expression significantly enriched (Figure 3D). The top 50 proteins with the most severe uptake defects showed some specificity and were significantly enriched for the tricarboxylic acid (TCA) cycle and respiratory electron transport and mitochondrial translation (Figure 3E). Overall, our data showed a range of different effects of CCCP on the uptake of individual proteins, with a considerable number of proteins not affected, while the large majority of proteins exhibited decreased uptake rates. Notably, the observed differences were not directly attributable to features such as protein function or localization within mitochondria.

### Stress shapes mitochondrial protein uptake via translation and import regulation

**Figure 4:**
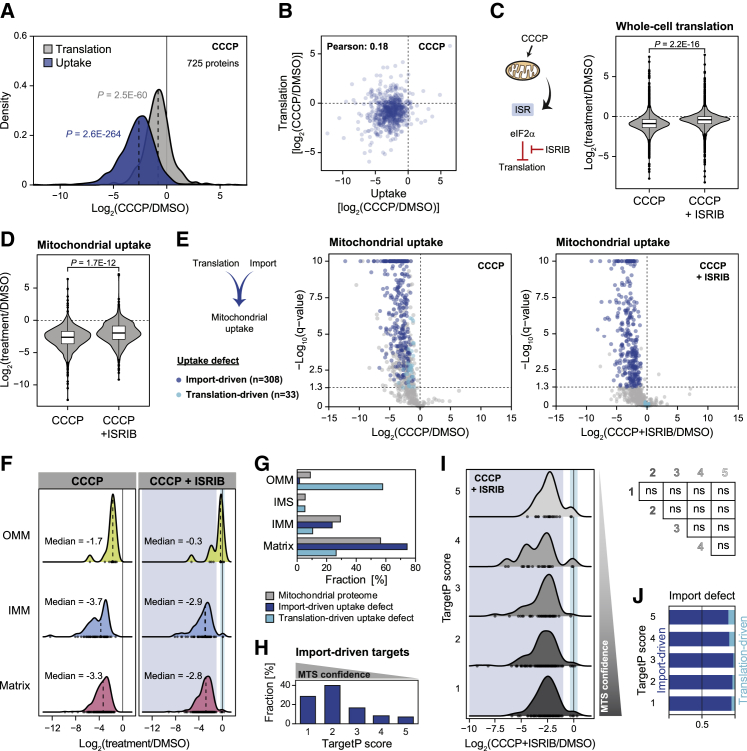
Stress shapes mitochondrial protein uptake via translation and import regulation (A) Density plot of fold changes of translation and mitochondrial uptake of mitochondrial proteins upon CCCP treatment compared to DMSO-treated control cells (n = 3). Dashed lines indicate median values of the distributions. Kolmogorov-Smirnov test against normal distribution ∼0, with standard deviation obtained for the tested distribution. (B) Correlation of CCCP-induced fold changes of protein translation and mitochondrial uptake, compared to DMSO-treated cells. (C) Scheme of integrated stress response (ISR) activation by CCCP and inhibition of the resulting translation attenuation by ISRIB (left). Violin plot of CCCP-induced changes in whole-cell translation with and without co-treatment with ISRIB, compared to DMSO-treated control cells (right, n = 3). Center line, median; box limits, upper and lower quartiles; whiskers, 1.5× interquartile range; points, outliers. Two-sample Kolmogorov-Smirnov test. (D) Violin plot of CCCP-induced mitochondrial protein uptake changes in the absence and presence of ISRIB, compared to DMSO-treated control cells (n = 3). Center line, median; box limits, upper and lower quartiles; whiskers, 1.5× interquartile range; points, outliers. Two-sample Kolmogorov-Smirnov test. (E) Targets with significant CCCP-induced uptake defects (fold change [log_2_] ≤ −1 and adjusted p ≤ 0.05) segregate into 2 groups with import-driven (308 proteins; fold change [log_2_] ≤ −1 and adjusted p ≤ 0.05) or translation-driven (33 proteins; −0.35 ≤ fold change [log_2_] ≤ 0.35) uptake defects upon co-treatment with ISRIB. Volcano plots of cells treated with CCCP (center) or CCCP and ISRIB (right). Adjusted p > 10 were set to p = 10 for plotting; original adjusted p values given in Table S5. (F) Density plots of changes in mitochondrial protein uptake upon CCCP (left) or CCCP+ISRIB (right) compared to DMSO-treated cells for import-driven or translation-driven proteins (from E) separated by suborganellar locations. Dashed lines indicate median values of the distributions. Dots at the bottom of the distributions indicate number and location of data points. Areas of targets with import- or translation-driven uptake defects are highlighted in blue and cyan, respectively. Please note that the dataset only contains 2 IMS proteins, which is why their distributions are not included in the figure. (G) Histogram showing the distribution of mitochondrial proteins and proteins with import- or translation-driven import defects. (H) Distribution of mitochondrial proteins with import-driven import defects across different TargetP scores. Increasing TargetP scores negatively correlate with mitochondrial targeting signal (MTS) confidence. (I) Density plot of changes in mitochondrial protein uptake upon treatment with CCCP and ISRIB of proteins with import- or translation-driven uptake defects (left). Proteins were grouped based on their TargetP score. Areas of targets with import- or translation-driven uptake defects were highlighted in blue and cyan, respectively. Dots at the bottom of the distributions indicate number and location of data points. Inequality of the distributions was determined with a 2-sample Kolmogorov-Smirnov test (right). ns, non-significant. (J) Bar graph showing the fraction of targets with import- or translation-driven uptake defects for each TargetP score. See also Figure S4 and Table S5.

We reasoned that the complex uptake changes observed upon CCCP treatment may be explained by activation of the ISR driven by CCCP-induced mitochondrial depolarization (Münch and Harper, 2016; Samluk et al., 2019; Fessler et al., 2020). Consequently, changes in protein uptake behavior observed for a range of mitochondrial stresses and disease-related conditions may be driven by two separate mechanisms (1) reduced activity of the import machinery and (2) by a decrease in mitochondrial protein translation also causing a decrease in uptake. To evaluate the effects of translation regulation on uptake, we characterized the effect of CCCP treatment on the translation of mitochondrial proteins and compared it to the observed uptake defects (Figure 4A; Table S5). While both translation and uptake were reduced, thus confirming that translation contributed to the significant reduction in protein uptake, correlation analysis of individual proteins showed only a weak association (Figure 4B). Hence, while ISR-mediated translation attenuation had a global impact on mitochondrial protein uptake upon CCCP treatment, its effects were not uniform and instead were protein specific.

With both processes—protein translation and import—as main contributors to mitochondrial protein uptake, we attempted to uncouple the effects of CCCP on mitochondrial protein import from the effects on translation. To do so, we prevented ISR-mediated translation changes by using the ISR inhibitor ISRIB (Sidrauski et al., 2015) to exclusively monitor the import-driven effects. As expected, co-treatment with ISRIB largely reversed CCCP-induced translation defects (Figure 4C; Table S5) and reduced the decrease in mitochondrial protein uptake (Figure 4D; Table S5). CCCP-induced import-driven effects did not correlate with protein properties such as length, hydrophobicity, charge, or copy number (Figure S4A). In addition, the degradation of precursor proteins by the proteasome only contributed to a minor extent, as observed by co-treatment with the proteasome inhibitor MG132 (Figure S4B). Evaluating the effect of ISRIB on mitochondrial proteins that exhibited significant uptake defects upon CCCP treatment revealed two distinct protein groups (Figure 4E): The first and larger group, containing ∼55% of proteins with significant uptake defects, exhibited only mild uptake alterations and retained significant uptake defects when rescuing protein translation by ISRIB. Thus, uptake of these proteins appeared to be predominantly driven by the import machinery rather than translation changes. However, uptake of the second group, containing ∼6% of the proteins, was rescued when adding ISRIB, revealing translation and not the mitochondrial import machinery to be responsible for changes in uptake upon CCCP treatment.

To gain a better understanding of the protein populations exhibiting import- or translation-driven uptake defects, we analyzed their submitochondrial localization. Both groups with import- or translation-driven uptake defects each contained one protein assigned to the IMS. While the remaining three subcompartments contained some proteins with import- or translation-driven uptake defects upon treatment with CCCP, the different compartments showed severe differences (Figure 4F); proteins of the OMM appeared to be controlled by translation, with ISRIB co-treatment reversing uptake defects. In contrast, ISRIB rescued the uptake of proteins into the IMM or matrix to a lesser extent, with the large majority of protein uptake defects driven by the import machinery and not translation (Figure 4F). A similar pattern was observed when analyzing the contribution of each mitochondrial subcompartment to the protein groups with import- or translation-driven uptake defects (Figure 4G). The majority of targets with translation-driven uptake defects were annotated OMM proteins, whereas the group with import-driven defects was predominantly constituted by IMM and matrix proteins.

We next assessed the relationship between uptake defects and a possible contribution of the mitochondrial targeting signals (MTS) for targets with import-driven uptake defects. Using the TargetP confidence score obtained from MitoCarta3.0 (Emanuelsson et al., 2000; Rath et al., 2021), we differentiated between 5 confidence levels, where 1 indicates the highest and 5 the lowest confidence in assigning an MTS. Import machinery-controlled proteins were predominantly assigned to the two highest TargetP confidence levels with a gradual decrease with declining MTS confidence (Figure 4H). Surprisingly, analysis of uptake rates of the individual TargetP score populations revealed that these did not differ significantly and that members of both groups, with import- and translation-driven uptake defects, were distributed across all assigned TargetP scores (Figure 4I). We then quantified the fractions of targets with import-driven and translation-driven defects for each TargetP score level to assess which type of defect prevails, depending on MTS confidence (Figure 4J). The uptake of proteins with a predicted MTS of any TargetP score was predominantly controlled on the import level; however, the fraction of proteins with translation-driven defects increased with declining MTS confidence. Hence, MTS confidence was a strong indicator of import machinery dependence but did not correlate with the extent of import-driven uptake defects.

### Mitochondrial uptake of respiratory complex I and mitochondrial translation machinery components is controlled by their import efficiency

**Figure 5:**
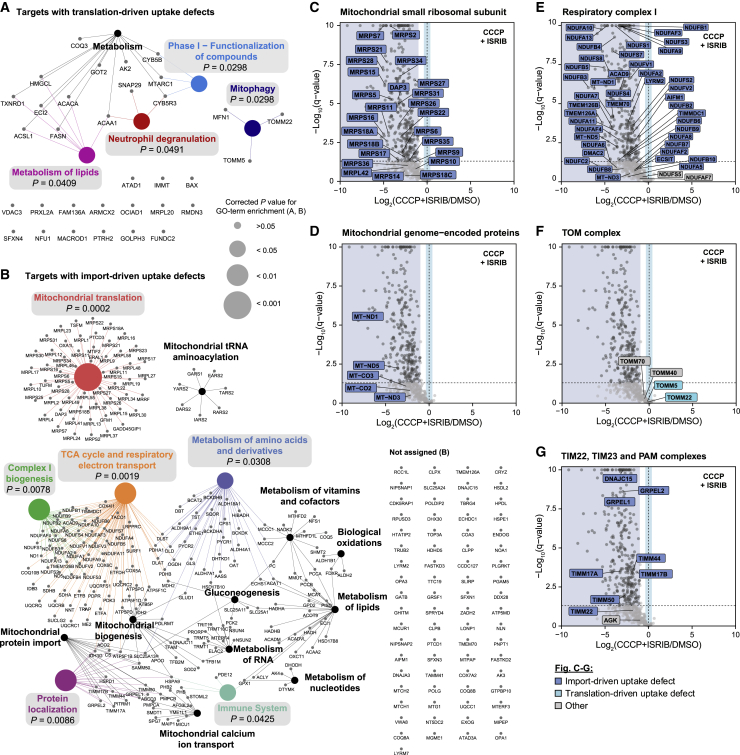
Mitochondrial uptake of respiratory complex I and mitochondrial translation machinery components is controlled by their import efficiency (A and B) Reactome pathway networks of targets with translation-driven (A) or import-driven uptake defects (B) upon CCCP treatment, prepared with the Cytoscape plug-in ClueGO. Significantly enriched pathways were highlighted with colors and gray boxes. p value corrected with Benjamini-Hochberg procedure. Full GO term lists are provided in Table S2. (C–G) Volcano plots showing fold changes of mitochondrial protein uptake plotted against the adjusted p value for cells co-treated with CCCP and ISRIB, compared to DMSO-treated control cells. Data points of components of the mitochondrial large ribosomal subunit (C), mitochondrial genome-encoded proteins (D), respiratory complex I (E), TOM complex (F), or TIMM22, TIM23, and PAM complexes (G) with significant CCCP-induced uptake defects (fold change [log_2_] ≤ −1 and adjusted p ≤ 0.05) were labeled and colored according to their uptake behavior. Data points of proteins changing significantly (fold change [log_2_] ≤ −1 or ≥ 1, and adjusted p ≤ 0.05) are shown in dark gray. Areas of targets with import- or translation-driven uptake defects were highlighted in blue and cyan, respectively. Note that only the fold change [log_2_] and not the adjusted p value upon CCCP+ISRIB treatment, was taken into account for classification of import- and translation-driven targets here. See also Tables S2 and S5.

To gain an overview of mitochondrial pathways predominantly driven by translation or import machinery upon CCCP treatment, we next carried out Reactome pathway-based network analyses of all identified targets. Proteins exhibiting translation-driven uptake defects were part of multiple significantly enriched pathways, such as mitophagy and metabolism of lipids (Figure 5A). The latter appeared in networks of both import- and translation-driven targets, indicating that uptake of functionally related proteins was not necessarily co-regulated (Figures 5A and 5B). Notably, several pathways, such as the TCA cycle and respiratory electron transport, complex I biogenesis, and mitochondrial translation were significantly enriched among import-driven targets, 2 of which had also been enriched among the top 50 targets with the most severe CCCP-caused uptake defects (Figures 3E and 5B). All of the components of the mitochondrial small ribosomal subunit with significant CCCP-induced uptake defects were regulated on the import level (Figure 5C). Accordingly, all significantly affected mitochondrial genome-encoded proteins, which are essential components of respiratory complexes, showed import-driven reduction (Figure 5D). As these proteins are synthesized in the mitochondrial matrix and hence do not require import, their declining levels are likely an indirect consequence of attenuated mitochondrial translation capacity and could hint at a connection between import capacity and translation in the matrix. Defective uptake of respiratory complex I components was also import driven, revealing that complex I quickly responds to changes in import efficiency (Figure 5E).

Multiple proteins contributing to mitochondrial protein import were among the targets with CCCP-induced uptake defects (Figures 5A and 5B). All significantly affected members of the OMM-resident translocase of the outer mitochondrial membrane (TOM) complex were targets with translation-driven import defects (Figure 5F), which is in agreement with the finding that OMM proteins were enriched among proteins with translation-driven uptake defects (Figure 4G). Strikingly, components of IMM-resident import machineries were differently controlled and showed import-driven uptake defects (Figure 5G). These observations suggest that adjustment of import machinery abundance represents one layer to regulate protein uptake rates upon mitochondrial stress. Abundance of the import machinery at the OMM was tightly coupled to cytosolic translation, whereas IMM transport complex abundances were additionally regulated by membrane potential and thus by mitochondrial fitness.

## Discussion

We introduced mePROD^mt^, which uses a mitochondria-selective booster channel as an effective approach to increase the detection of newly synthesized mitochondrial proteins. The composition of the booster channel is highly flexible. Thus, this method can be readily adjusted to improve the sensitivity for other protein pools of choice. We showed that this procedure can also be applied to purified subcellular fractions (e.g., to study protein import). The improved sensitivity for mitochondrial proteins enabled a quantitative analysis of protein uptake of several hundred mitochondrial proteins simultaneously. Thus, this approach allows the characterization of mitochondrial protein uptake in a comprehensive manner from living cells without the necessity of protein tags, making it a powerful alternative to conventional import assays. The mitochondria-specific booster channel increased the sensitivity such that labeling times as short as 15 min were sufficient and therefore allowed kinetic studies. Analysis of this kinetics dataset provided uptake rates of imported proteins. Our data showed that uptake rates of components of functional complexes can vary strongly, as previously described for mitochondrial ribosomal complexes and respiratory chain complexes (Bogenhagen et al., 2018; Bogenhagen and Haley, 2020). Moreover, we were able to validate half-life measurements of short-lived proteins, such as NDUFS6, MCL1, and CHCHD2 (Burstein et al., 2018; Chen et al., 2019; Szczepanowska and Trifunovic, 2021).

The short labeling time required for mePROD^mt^ further enabled analysis of how protein uptake is acutely affected by CCCP-induced mitochondrial stress and provides the missing link between available transcriptome and proteome data (Quirós et al., 2017). Strikingly, we found that the effect of CCCP on protein uptake was not uniform for all detected mitochondrial proteins. We demonstrate that CCCP-induced mitochondrial stress results in a broad decline of mitochondrial protein uptake, likely contributing to the downregulation of mitochondrial protein abundance as reported by Quirós et al. (2017). We showed that declining protein uptake was not caused by enhanced proteasomal degradation. Major lysosomal degradation effects are also unlikely, since HeLa cells lack a functional mitophagy machinery and treatments occurred acutely (Xiao et al., 2017; Villa et al., 2017).

We identified several mitochondrial proteins with unchanged uptake rates. Whether these proteins harbor a beneficial function during mitochondrial stress and how uptake of these proteins is favored over uptake of the majority of mitochondrial proteins requires future study. Our data revealed that proteins required for mitochondrial translation and respiratory electron transport are among those with the most severe CCCP-induced uptake defects. This agrees with studies reporting that mitochondrial stress leads to decreased levels of mitochondrial ribosomal proteins and proteins of the respiratory chain (Zhu et al., 2012; Quirós et al., 2017; Franco-Iborra et al., 2018).

Our data are consistent with the observation that translation attenuation makes a considerable contribution to the changing mitochondrial proteome upon CCCP-induced mitochondrial stress (Quirós et al., 2017). Beyond that, we confirmed protein import as an additional layer of regulation that controls protein uptake during stress. We showed that uptake of certain mitochondrial proteins, among which OMM proteins were enriched, was solely regulated by their translation rate upon CCCP treatment. By contrast, uptake of the majority of detected mitochondrial proteins, which predominantly contained IMM and matrix proteins, was affected on the levels of both translation and import efficiency. Among the targets with import-driven uptake defects were proteins involved in the TCA cycle and respiratory electron transport, mitochondrial translation, and mitochondrial protein import at the inner membrane. These machineries are functionally interconnected (O’Brien, 2003; van der Laan et al., 2006; Kulawiak et al., 2013; Gopisetty and Thangarajan, 2016; Schendzielorz et al., 2017; Cogliati et al., 2018) and have been shown to be targets of mitochondrial stress responses to adapt mitochondrial protein content and function (Zhu et al., 2012; Rainbolt et al., 2013; Quirós et al., 2017; Franco-Iborra et al., 2018; Priesnitz and Becker, 2018).

The comprehensive nature of the proteomics-based uptake assay makes it a promising tool to investigate key aspects of mitochondrial protein uptake, such as defining the substrate spectrum of the individual import pathways and assessment of how altered protein uptake reshapes the mitochondrial proteome when mitochondrial function is compromised. As we showed, translation regulation makes an underestimated contribution to stress-induced adaptation of mitochondrial protein uptake. The underlying causes of impaired uptake (i.e., the relevance of translation attenuation) in disease contexts have not been sufficiently addressed yet. Adjusting the abundance and activity of mitochondrial import machineries to regulate protein uptake is a common theme upon treatment with multiple mitochondrial stressors (Rainbolt et al., 2013; Priesnitz and Becker, 2018). We found that components of the translocation machineries themselves are among the targets with reduced uptake, showing that translocation machinery abundance upon stress is regulated by a decreased influx of newly synthesized subunits. The proper regulation of mitochondrial protein import is essential for cellular health, and deregulated protein import has been demonstrated in various diseases, such as cancer, Parkinson’s disease (PD), and Huntington’s disease (HD) (Kang et al., 2018; Nicolas et al., 2019). For instance, PD-associated forms of α-synuclein were shown to affect mitochondrial protein import by physically interacting with TOM20 (Di Maio et al., 2016). Likewise, HD-associated mutant huntingtin interferes with import at the IMM by interaction with TIM23 (Yano et al., 2014). Wide-ranging analyses of the consequences of impaired import on mitochondrial protein uptake, and thereby the mitochondrial proteome, may provide valuable new insights about the cellular foundation of disease phenotypes.

### Limitations of the study

mePROD^mt^ enables mitochondrial protein import measurements from cells, without the need of overexpression of candidate proteins or mixing purified mitochondria with candidate proteins. While being able to detect and quantify hundreds of mitochondrial proteins, mePROD^mt^ cannot cover the complete mitochondrial proteome. Like other proteomics methods, it remains limited by the abundance of mitochondrial proteins and MS compatibility of their resulting peptides. The booster peptides largely improve the sensitivity for low-abundance proteins, specifically in their newly synthesized form, but do not improve their physical properties. Newly synthesized proteins are labeled metabolically, which requires labeling in living cells. Consequently, mePROD^mt^ cannot be carried out with non-labeled samples, and the experimental system needs to be amendable to culturing with the isotopically labeled amino acids. Absolute quantification of the abundance or uptake of proteins is not possible. Instead, mePROD^mt^ carries out relative quantification that allows direct comparison of different time points or conditions within one multiplex.

## STAR★Methods

### Key resources table

**Table:** 

REAGENT or RESOURCE	SOURCE	IDENTIFIER
**Chemicals, peptides, and recombinant proteins**		

anti-ACADSB, 1/1000	Proteintech	Cat#13122-AP; RRID:AB_10638904
anti-ACTIN, 1/4000	SantaCruz	Cat#sc69879; RRID:AB_1119529
anti-ATP5A1, 1/1000	Proteintech	Cat#14676-1-AP; RRID:AB_2061761
anti-caspase-9, 1/1000	Cell signaling	Cat#9502S; RRID:AB_2068621
anti-cleaved caspase-3, 1/1000	Cell signaling	Cat#9664T; RRID:AB_2070042
anti-cleaved PARP1, 1/2000	Cell signaling	Cat#5625T; RRID:AB_10699459
anti-COX16, 1/1000	Proteintech	Cat#19425-1-AP; RRID:AB_10666854
anti-COX5B, 1/1000	Proteintech	Cat#11418-2-AP; RRID:AB_2245376
anti-COXIVL1, 1/1000	Proteintech	Cat#66110-1-Ig; RRID:AB_2881509
anti-GRPEL2, 1/1000	Proteintech	Cat#17751-1-AP; RRID:AB_2878432
Anti-HaloTag 1/1000	Promega	Cat# G9211; RRID:AB_2688011
anti-HSPA9, 1/1000	Abcam	Cat#ab2799; RRID:AB_303311
anti-HSPD1, 1/1000	Abcam	Cat#ab46798; RRID:AB_881444
anti-ME3, 1/1000	Abcam	Cat#ab172972
anti-SDHA, 1/1000	Proteintech	Cat#14865-1-AP; RRID:AB_11182164
anti-TIMM23, 1/1000	Proteintech	Cat#11123-1-AP; RRID:AB_615045
Arginine 10	Cambridge Isotope Laboratories	Cat#CNLM-539-H-PK
CCCP	Sigma-Aldrich	Cat#C2759
HaloTag Biotin Ligand	Promega	Cat#G8281
HaloTag Ligand empty	N/A	N/A
IRDye 680RD Donkey anti-mouse, 1/15000	Li-Cor	Cat#926-68072
IRDye 800CW Donkey anti-rabbit, 1/15000	Li-Cor	Cat#926-32213
IRDye 800CW Streptavidin 1/1000-1/5000	Li-Cor	Cat# 926-32230
ISRIB	Sigma-Aldrich	Cat#SML0843
Lysine 8	Cambridge Isotope Laboratories	Cat#CNLM-291-H-PK
MG-132	Sigma-Aldrich	Cat#M7449
MitoBloCk-6	Focus Biomolecules	Cat#10-1472
Mitotracker Red FM	Thermo Fisher Scientific	Cat#M22425
Staurosporin	Sigma-Aldrich	Cat#S5921-.1MG
TMT reagents	Thermo Fisher Scientific	Cat#A34808, Cat#A44520

**Critical commercial assays**		

μBCA microplate assay	Thermo Fisher Scientific	Cat#23235
Pierce High pH Reversed-Phase Peptide Fractionation Kit	Thermo Fisher Scientific	Cat#84868

**Deposited data**		

MS raw data: Human mitochondrial protein import proteomics	ProteomeXchange Consortium	PRIDE: PXD022521
MS raw data: Human mitochondrial protein import proteomics - Proteinase K control	ProteomeXchange Consortium	PRIDE: PXD024066
MS raw data: Human mitochondrial protein import proteomics LFQ	ProteomeXchange Consortium	PRIDE: PXD024054
Western blot and imaging data	Mendeley	Mendeley Data: https://data.mendeley.com/datasets/68pdjd5m53/2
Original code for DynaTMT	Zenodo	Zenodo: https://zenodo.org/record/5575143
Original code for DynaTMT-py	Zenodo	Zenodo: https://zenodo.org/record/5575124

**Experimental models: cell lines**		

HeLa	ATCC	N/A

**Recombinant DNA**		

HaloTag	An et al., 2020	N/A
MTS-EGFP (Su9-EGFP)	Chen et al., 2003	Addgene #23214
pHAGE C-TAP	gift of Richard Mulligan (Harvard Medical School, Boston, MA)	N/A

**Software and algorithms**		

ClueGo + Cluepedia v1-v2	Bindea et al., 2009, 2013	https://academic.oup.com/bioinformatics/article-lookup/doi/10.1093/bioinformatics/btt019, https://academic.oup.com/bioinformatics/article-lookup/doi/10.1093/bioinformatics/btt019
CQ1 Software	Yokogawa	N/A
Prism 6	GraphPad	https://graphpad-prism.software.informer.com/6.0/
Cytoscape 3.5.1	Shannon et al., 2003	https://cytoscape.org/
ggpubr	Kassambara, 2020	https://cran.r-project.org/web/packages/ggpubr/index.html(2020)
ggrepel	Slowikowski, 2020	https://cran.r-project.org/web/packages/ggpubr/index.html
ggridges	Wilke, 2020	https://cran.r-project.org/web/packages/ggridges/index.html
ImageJ 1.53c	Schneider et al., 2012	http://www.nature.com/articles/nmeth.2089
MaxQuant 1.6.17	Cox and Mann 2008	http://www.nature.com/articles/nbt.1511
Proteome Discoverer 2.4	Themo Fisher Scientific	Cat#OPTON-30957
Python 3.6	Python Consortium	https://www.python.org/
statsmodels 0.12.2	Seabold and Perktold, 2010	https://pypi.org/project/statsmodels/
SciPy	Virtanen et al., 2020	http://www.nature.com/articles/s41592-019-0686-2
RStudio version 3.6.1	R Core Development Team, 2019	https://www.r-project.org/
tidyverse	Wickham et al., 2019	https://joss.theoj.org/papers/10.21105/joss.01686
Tune 2.9	Thermo Fisher Scientific	N/A
Xcalibur 4.0	Thermo Fisher Scientific	Cat#OPTON-30965
DynaTMT(-py)	This study	GitHub: https://github.com/klannk/DynaTMThttps://github.com/klannk/DynaTMT-py

**Other**		

Orbitrap Fusion Lumos Tribrid MS	Thermo Fisher Scientific	Cat#IQLAAEGAAPFADBMBHQ
QExactive HF Orbitrap MS	Thermo Fisher Scientific	Cat#IQLAAEGAAPFALGMBFZ

### Resource availability

#### Lead contact

Further information and requests for resources and reagents should be directed to and will be fulfilled by the Lead Contact, Christian Münch (ch.muench@em.uni-frankfurt.de).

#### Materials availability

This study did not generate new unique reagents.

### Experimental model and subject details

#### Cell lines

Human epithelial cervix-adenocarcinoma (HeLa) cells (female) were cultured in RPMI1640 medium (GIBCO 21875034) supplemented with 10% heat inactivated, sterile, non-dialyzed FBS (GIBCO 10270-106) in a humidified incubator at 37°C with 5% CO_2_. For SILAC labeling, cells were seeded in 10 cm plates in regular culture medium with a density of approximately 2x10^6^ cells per plate and grown over night. For treatment with CCCP and SILAC labeling, cells were washed twice with prewarmed PBS and treated for 2 h with 10 μM CCCP (Sigma-Aldrich C2759), with or without additional 500 nM ISRIB (Sigma-Aldrich SML0843), or with or without additional 10 μM MG-132 (Sigma-Aldrich M7449), in SILAC medium consisting of RPMI160 medium for SILAC (GIBCO 88365) supplemented with 100 μg/mL Arg10 (Cambridge Isotope Laboratories), 100 μg/mL Lys8 (Cambridge Isotope Laboratories) and 10% non-dialyzed FBS. For treatment with MitoBloCK-6 and SILAC labeling, cells were washed twice with prewarmed PBS and treated with 50 μM MitoBloCK-6 (Focus Biomolecules 10-1472) in regular culture medium for 4 h, followed by treatment with 50 μM MitoBloCK-6 in SILAC medium for another 2 h. Whole-cell and mitochondrial boosters were prepared from cells permanently cultured in SILAC medium for 3-4 weeks. For the noise channel, cells were cultured in regular culture medium. Cell line was purchased from ATCC, but not additionally authenticated.

### Method details

#### Constructs and cloning

HaloTag fusion constructs were cloned into the lentiviral overexpression vector pHAGE C-TAP (gift of Dr. Richard C. Mulligan, Harvard Medical School, Boston, MA). Human gene of interest sequences were amplified from HeLa ATCC cDNA and the RPL28-HaloTag7 plasmid from An et al. (2020) was used for the HaloTag sequence. Gene of interest- HaloTag fusion genes were integrated with Gibson cloning in pHAGE C-TAP in between EcoRI and *NotI* restriction sites.

#### Cell harvest, lysis and mitochondrial isolation

After treatment and SILAC labeling, cells were detached with 0.25% Trypsin (GIBCO 25200056) and washed twice with cold PBS. Pellets were snap-frozen in liquid nitrogen and stored at −80°C. Mitochondria were isolated from cells as described before (Bozidis et al., 2007). Briefly, cell pellets were resuspended in cold MTE buffer containing 0.27 M D-mannitol, 0.01 M Tris-base, 0.1 mM EDTA and one protease inhibitor cocktail tablet (EDTA-free, Roche). Cell suspensions were sonicated with a Sonic Vibra Cell at 10 s ON/ 10 s OFF pulse for 60 s at a maximal amplitude of 25% to disrupt the cell membrane and shear genomic DNA. After sonication, 10% (v/v) of each sample was separated for measuring translation of mitochondrial proteins. The remaining volume was centrifuged at 1,400 × g for 10 min at 4°C. The supernatant was then centrifuged at 15,000 × g for 10 min at 4°C. The obtained pellet containing mitochondria was washed once with MTE buffer and spun down at 15,000 × g for 5 min at 4°C. All samples (for measuring translation and uptake) were denatured with 2% SDS, 50 mM Tris-HCl pH 8, 150 mM NaCl, 10 mM TCEP, 40 mM chloroacetamide and protease inhibitor cocktail tablet (EDTA-free, Roche) at 95°C for 10 min.

#### Proteinase K digestion

Mitochondrial isolates were washed twice by resuspension in 1 mL cold MTE buffer and pelleting at 15,000 × g, 4°C for 10 min to remove protease inhibitors. Pellets were taken up in 500 μL MTE buffer containing 500 μg/mL proteinase K (QIAGEN, 19131) and incubated on ice for 30 min. Mitochondria were pelleted and washed twice with MTE buffer to remove digested proteins and proteinase K.

#### Sample preparation for LC-MS/MS

Lysates were prepared for LC-MS/MS as previously described (Klann et al., 2020). Briefly, pure proteins were obtained with methanol/chloroform precipitation. Protein pellets were then resuspended in 8 M Urea, 10 mM EPPS pH 8.2 and protein concentrations were determined with a BCA assay (ThermoFisher Scientific 23225). Approximately 20 μg of protein was digested overnight at 37°C with LysC (Wako Chemicals) at 1:50 (w/w) ratio and Trypsin (Promega V5113) at 1:100 (w/w) ratio. Peptides were then purified using Empore C18 (Octadecyl) resin material (3M Empore). Peptide concentrations were determined with a μBCA assay (ThermoFisher Scientific 23235) and 10 μg of peptide per sample was labeled with 11 plex (Thermo Scientific, A34808) or 16 plex (Thermo Scientific, A44520) TMT reagents. TMT labeled samples were adjusted to equal amounts (except boost channel). Adjustment was assessed by a LC-MS test run with small sample amount, before fractionation of the total sample. Labeled peptide samples were pooled, fractionated into 8 fractions using the High pH Reversed-Phase Peptide Fractionation Kit (ThermoFisher Scientific 84868) according to the manufacturer protocol and dried. Additionally, for label free single shots, 10 μg of peptide is cleaned up with Empore C18 stage tipping and dried right away for shooting.

#### Mass spectrometry

One μg of dried peptides of each fraction was resuspended in 2% (v/v) acetonitrile / 1% (v/v) formic acid solution. Samples were shot with settings described in Klann and Münch (2020). Briefly, peptides were separated with Easy nLC 1200 (ThermoFisher Scientific) using a 30 cm long, 75 μm inner diameter fused-silica column packed with 1.9 μm C18 particles (ReproSil-Pur, Dr. Maisch) and kept at 50°C using an integrated column oven (Sonation). Individual peptides were eluted by a non-linear gradient from 5 to 40% B over 120 min for fractionated samples (210 min for fractionated CCCP samples) or 210 min for single shots, followed by a stepwise increase to 95% B in 6 min, which was kept for another 9 min and sprayed into an Orbitrap Fusion Lumos Tribrid Mass Spectrometer (ThermoFisher Scientific). Full scan MS spectra (350-1,400 m/z) were acquired with a resolution of 120,000 at m/z 100, maximum injection time of 100 ms and AGC target value of 4 × 10^5^. For targeted mass difference (TMD) based runs, the 10 most intense ions with a charge state of 2-5 were selected together with their labeled counterparts (Targeted Mass Difference Filter, Arg and lysine delta mass, 5%–100% partner intensity range with 7 ppm mass difference tolerance), resulting in 20 dependent scans (Top20). For data dependent acquisition (DDA) based runs (Figures S1B and S1C), the 20 most intense peptides with a charge state between 2 and 5 per full scan were isolated (Top20). Precursors were isolated in the quadrupole with an isolation window of 0.7 Th. MS2 scans were performed in the quadrupole using a maximum injection time of 86 ms, AGC target value of 1 × 10^5^. Ions were then fragmented using HCD with a normalized collision energy (NCE) of 35% and analyzed in the Orbitrap with a resolution of 50,000 at m/z 200. Repeated sequencing of already acquired precursors was limited by setting a dynamic exclusion of 60 s and 7 ppm, and advanced peak determination was deactivated.

For label free quantification, individual peptides were eluted by a non-linear gradient from 4 to 40% B over 210 min, followed by a stepwise increase to 95% B in 6 min, which was kept for another 9 min and sprayed into a QExactive HF mass spectrometer (ThermoFisher Scientific). Full scan MS spectra (300-1,650 m/z) were acquired with a resolution of 60,000 at m/z 200, maximum injection time of 20 ms and AGC target value of 3 × 10^6^. The 15 most intense precursors were selected for fragmentation (Top 15) and isolated with a quadrupole isolation window of 1.4 Th. MS2 scans were acquired in centroid mode with a resolution of 15,000 at m/z 200, a maximum injection time of 25ms, AGC target value of 1 × 10^5^. Ions were then fragmented using higher energy collisional dissociation (HCD) with a normalized collision energy (NCE) of 27; and the dynamic exclusion was set to 20 s to minimize the acquisition of fragment spectra of already acquired precursors.

#### MTS-EGFP protein import assay

HeLa cells were transiently transfected with the MTS-EGFP plasmid (gift from David Chan, Addgene # 23214) for 24 h (Chen et al., 2003). After 4 h of transfection, medium was exchanged and cells were treated with 10 μM CCCP (Sigma-Aldrich, C2759) or DMSO overnight. Cells were stained with 200 nM Mitotracker Red FM (Thermo Fisher Scientific, M22425) for 30 min prior to imaging, washed once with 1x PBS and incubated in medium during live imaging on a Yokogawa CQ-1 microscope. Images were acquired at 488 nm excitation and 525/50 nm emission for GFP, and 561 nm excitation and 685/40 nm emission for Mitotracker Red FM. One-hundred cells per biological replicate were manually categorized in one of four categories to assess the efficiency of mitochondrial protein import of MTS-EGFP, using ImageJ 1.53c (Schneider et al., 2012).

#### Immunoblotting

Cell pellets were lyzed with RIPA Buffer (Sigma R0278) containing protease inhibitor cocktail and benzonase (Millipore E1014) on ice for 20 minutes. The cell lysates were spun at 15,000xg at 4°C for 10 minutes. Supernatants were transferred to fresh tubes. Protein amounts were measured using BCA protein assay kit. 20-30 μg of proteins were mixed with sample loading dye, boiled at 95°C for 5 mins and loaded on precast polyacrylamide gels. The Chameleon duo pre-stained ladder (Li-Cor 928-60000) was used to detect size of the proteins for near-infrared detection. For larger proteins (> 40kDa) 12% SDS gels (Invitrogen Bolt NW00122BOX) and for smaller proteins (< 40kDa) 16% SDS gel (Invitrogen Novex EC66952BOX) were used for protein separation. To detect the precursor forms of mitochondrial proteins, proteins were run for 1 hour at 80V and 2 hours at 120V. For other separations, proteins were run at 180V for 1 hour. Gels were transferred to 0.2 μm nitrocellulose membrane using mini transfer pack (Bio-Rad 1704158) with trans-blot turbo transfer system (Bio-Rad 1704150) for 7 minutes. Membranes were blocked with 5% BSA (Sigma) for 1 hour at room temperature. Primary antibodies; HSPD1 (Abcam ab46798, 1/1000), ATP5A1 (Proteintech 14676-1-AP, 1/1000), HSPA9 (Abcam ab2799, 1/1000), SDHA (Proteintech 14865-1-AP, 1/1000), COX5B (Proteintech 11418-2-AP, 1/1000), COXIVL1 (Proteintech 66110-1-Ig, 1/1000), TIMM23 (proteintech 11123-1-AP, 1/1000), COX16 (Proteintech 19425-1-AP, 1/1000), GRPEL2 (Proteintech 17751-1-AP, 1/1000), ACADSB (Proteintech 13122-AP, 1/1000), ME3 (Abcam ab172972, 1/1000), cleaved PARP1 (Cell signaling 5625T, 1/2000), caspase-9 (Cell signaling 9502S, 1/1000), cleaved caspase-3 (Cell signaling 9664T, 1/1000), ACTIN (SantaCruz sc69879, 1/4000) in 5% BSA in PBS were incubated at 4 oC for overnight. Membranes were washed 3 times for 5 minutes with PBS-T (0.1% Tween). Secondary antibodies; IRDye 680RD Donkey anti-mouse (Li-Cor 926-68072, 1/15000) and IRDye 800CW Donkey anti-rabbit (Li-Cor 926-32213, 1/15000) were used in PBS-T and incubated for 1 hour in the dark. Membranes were washed 3 times for 5 minutes with PBS-T, followed by 1 wash with PBS. Membranes were imaged using an Odyssey CLx imager (Li-Cor) and images were analyzed with image studio lite v5.2 (Li-Cor).

#### Stable HaloTag-protein cell line generation

Lentiviral particles were generated in HEK293T cells by transfection with pHAGE HaloTag fusion vectors, containing a mitochondrial gene of interested cloned in frame with HaloTag7. In addition, following helper vectors were co-transfected: pHDM-VSVG, -HGPM2, -tatIB and pRC-CMV-revIB. Lipofectamine2000 (Thermo Fisher Scientific, 11668019) was used with Opti-MEM I (Thermo Fisher Scientific, 31985-047) according to manufacturer protocol, including a medium exchange after 6 h. Lentiviral particle containing supernatant was harvested 48 h after transfection, subjected to centrifugation at 1000xg for 3 min and added 1/10 together with 8 μg/ml polybrene (Sigma, H9268) to HeLa cells. HaloTag fusion-positive cells were selected by addition of 2 μg/ml puromycin for 11 days. Each cell line was checked by TMR labeling and fluorescence microscopy for correct mitochondrial localization of the HaloTag-fusion protein.

#### HaloTag-protein uptake assay

Cells stably expressing HaloTag-fusion protein were seeded in 10-cm dishes and, on the same day of seeding and after cells had attached, 5 μM HaloTag® empty Ligand in 5 mL RPMI 10%FBS medium was added to the cells overnight. This saturated all previous synthesized HaloTag fusion protein. The next morning cells were washed twice with prewarmed PBS (37°C) for 1 min each, and once with prewarmed RPMI 10% FBS medium for 5 min at 37°C. Two more 10 min-washes with prewarmed RPMI 10% FBS medium with 0.1% DMSO or 10 μM CCCP were done. This started the treatment time. For the last hour of the treatment 5μM HaloTag® Biotin Ligand (in 2 ml) was added to the cells to label during the treatment synthesized HaloTag fusion protein. After 5:50 h treatment the cells were washed twice for 1 min with 1x PBS (37°C) and once for 10 min with 0.1% DMSO or 10 μM CCCP-containing 10% FBS medium. The cells were harvested by 1 mL 0.25% Trypsin/EDTA and resuspended in 7 mL 4°C 10% FBS RPMI medium. Then, the cells were washed twice with ice-cold 1x PBS, pelleted by 800xg for 5 min and transferred into 2 mL low binding tubes.

Mitochondria were isolated from cells as described before (Bozidis et al., 2007). Briefly, cell pellets were resuspended in 1ml cold MTE buffer containing 0.27 M D-mannitol, 0.01 M Tris-base, 0.1 mM EDTA and one protease inhibitor cocktail tablet per 10 mL (EDTA-free, Roche). Cell suspensions were sonicated with a Sonic Vibra Cell at 10 s ON/ 10 s OFF pulse for 60 s at a maximal amplitude of 25% to disrupt the cell membrane and shear genomic DNA. The sample was spun at 1,400 × g for 10 min at 4°C. The supernatant was then spun at 15,000 × g for 10 min at 4°C. The obtained pellet containing mitochondria was washed once with MTE buffer and spun down at 15,000 × g for 5 min at 4°C. All samples were denatured with 2x SDS-loading buffer and protease inhibitor cocktail tablet (EDTA-free, Roche) at 95°C for 5 min. The mitochondrial imported proteins were analyzed via immunoblotting against biotinylated proteins utilizing Streptavidin-800 (Li-Cor) and compared to total HaloTagged protein via antiHaloTag (Promega) immunodetection and antiMouse-680 nm (Li-Cor).

### Quantification and statistical analysis

#### Processing of raw data

Raw data of TMD samples was analyzed with Proteome Discoverer (PD) 2.4 (ThermoFisher Scientific) and SequenceHT node was selected for database searches. Human trypsin digested proteome (*Homo sapiens* SwissProt database [TaxID:9606, 2018-11-21]) was used for protein identifications. Contaminants (MaxQuant “contamination.fasta”) were determined for quality control. TMT6 (+229.163) for TMT 11 plex and TMTpro (+304.207) for TMT 16 plex at the N terminus and carbamidomethyl (+57.021) at cysteine residues were set as fixed modifications. TMT6 (K, +229.163), TMT6+K8 (K, +237.177), Arg10 (R, +10.008) for TMT 11 plex, and TMTpro (K, +304.207), TMTpro+K8 (K, +312.221), Arg10 (R, +10.008) for TMT 16 plex, and methionine oxidation (M, +15.995) as well as Met-loss + Acetyl (M, −89.030) at the protein N terminus were set for dynamic modifications. Precursor mass tolerance was set to 10 ppm and fragment mass tolerance was set to 0.02 Da. Default Percolator settings in PD were used to filter perfect spectrum matches (PSMs). Reporter ion quantification was achieved with default settings in consensus workflow. PSMs were exported for further analysis (Klann and Münch, 2020) using the DynaTMT package (this study, detailed below).

Raw data of DDA samples without SILAC labeling was analyzed with Proteome Discoverer (PD) 2.4 (ThermoFisher Scientific) with the same parameters used for TMD samples, except that the step of heavy peptide extraction was omitted.

Raw files for label free single shots were analyzed with MaxQuant 1.6.17 with default settings using human trypsin digested proteome (*Homo sapiens* SwissProt database [TaxID:9606, version 2020-03-12]) (Cox and Mann, 2008). Carbamidomethyl fixed modification and acetyl and methionine oxidation dynamic modifications were used. For each protein, from mitochondrial or whole cell proteome, an intensity-based absolute quantification (iBAQ) (Schwanhäusser et al., 2011) was used as a measure of protein abundance.

Human MitoCarta3.0 was used for annotation of mitochondrial proteins (Rath et al., 2021). Submitochondrial localizations were annotated based on MitoCarta3.0 in combination with information retrieved from Uniprot, since the pool of annotated IMM proteins in MitoCarta3.0 contains peripheral membrane proteins, which should be handled as IMS or matrix proteins for protein uptake studies.

#### DynaTMT

The DynaTMT application and package include all relevant steps of pSILAC data analysis described previously and bundles them in an easy-to-use format, compatible with different inputs and pipeline frameworks (Klann and Münch, 2020; Klann et al., 2020).

#### Injection time adjustment

To adjust TMT intensities for their injection time (Klann and Münch, 2020), the TMT abundances are divided by the injection times by the following formula, where “i” is the TMT channel:

$$Intensity_{adjusted,\;i}=\;\frac{Intensity_{raw,i}}{Ion\;injection\;time}*1,000$$

#### Normalization

Three different modes can be used for normalization: 1) Total intensity normalization uses the summed intensity per TMT channel to calculate the normalization factors relative to the channel with the lowest total intensity. 2) Median normalization uses the median intensity of the channels instead of the sum. 3) TMM is a python implementation of the trimmed mean of M-values normalization introduced by Robinson and Oshlack (2010). We used total intensity normalization throughout this manuscript.

#### Baseline correction and protein quantification rollup

The baseline correction for mePROD experiments is performed as described before (Klann et al., 2020): On peptide or perfect spectrum match (PSM) level (according to input), the abundance of the noise channel, which contains a sample that is not SILAC labeled, will be subtracted from all other samples (Klann et al., 2020). This intensity is assumed to be generated from co-fragmented light peptides and can be considered as noise. To avoid artifacts generated by very small remaining intensities, we implemented a threshold value of 5 on average over all channels. After baseline correction, the mean of the channels has to be greater than the threshold value to be considered for further quantification. Otherwise, the peptide will be excluded. This threshold value can be fine-tuned empirically and set in the DynaTMT-py package. Negative intensities after correction will be set to zero to avoid negative numbers for noise measurements (the python package provides the possibility to change this behavior to either using random numbers between zero and one or keep the negative values).

For the DynaTMT browsertool, protein rollup is performed by building the sum of all peptides/PSMs for a given unique protein identifier as this is the most implemented method in processing softwares like the Proteome Discoverer (PD). In the python package, the method can be set to ‘sum’, ‘mean’ or ‘median’ to calculate protein quantifications. In addition, the tool provides with peptide level tables as intermediate output to facilitate other downstream analysis. We used summing all peptides throughout the whole study.

#### DynaTMT browsertool

The DynaTMT browsertool was built using HTML, Jinja2, JavaScript and Python 3.8. Using the FLASK package, a python server starts processing and routing of the application. The server then uses the DynaTMT python package to process the input files. In addition to the DynaTMT package, the application uses the following packages: Python: Flask 1.1.2, Jinja2 2.11.2, numpy 1.19.4, pandas 1.1.4, pyinstaller 4.1, scipy 1.5.4, Werkzeug 1.0.1; JavaScript: Danfo 0.1.2, jquery 3.5.1, bootstrap 4.5.3, plotly.

The application in its distributed form does not need any pre-installed packages or programs, except a web browser like Firefox or Chrome. It is available from Zenodo (Zenodo: https://zenodo.org/record/5575143) and GitHub (GitHub: https://github.com/klannk/DynaTMT) together with its source code and compiled versions for Windows, MacOS, and Linux.

#### DynaTMT-py

The python package implementation of DynaTMT is available via Zenodo (Zenodo: https://zenodo.org/record/5575124), GitHub (GitHub: https://github.com/klannk/DynaTMT-py) and the PyPi repository (https://pypi.org/project/DynaTMT-py/) for python packages and is easily installable via the python package manager pip. It is intended to be implemented in already established pipelines and has some additional parameters that can be changed during analysis. The package is available under the GNU GPL v3 license.

#### Data analysis and statistics

N represents the number of biological replicates and is stated in the figure legends; except for Figure 4E, where n represents the number of proteins with import- or translation-driven uptake defects. Adjusted *P values* ≤ 0.05 were considered significant. Cutoffs for significant log_2_ fold changes were set to ± 0.7 for MitoBloCK-6 and ± 1 for CCCP as indicated in the figure legends.

##### Calculation of protein uptake slopes

To calculate mitochondrial uptake slopes, the increase in heavy peptide intensity over time was fitted for each protein using a linear least-square regression model with SciPy (Virtanen et al., 2020). The linear model was fitted using a concatenated fit over all replicates (n = 2). Slopes and R^2^ values were extracted and the latter used for quality control. The slopes were interpreted as relative uptake efficiencies, filtered for R^2^ values > 0.95 and grouped into four quantiles. For each quartile, the average slope was plotted, together with the overall median slope (Figure 2E). Protein half-lives were calculated from light peptides of the same dataset by one phase decay nonlinear regression analysis with 0.9 fitness cut-off (Stewart et al., 2011; Mathieson et al., 2018).

##### Calculation of protein copy numbers

Protein copy numbers were estimated using the ProteomicRuler plugin in perseus 1.6.15.0. Protein intensities derived from label-free quantification in the whole cell proteome sample were normalized by their molecular weight (Wiśniewski et al., 2014). Detectability correction was performed using the number of theoretical peptides created by trypsin digestion. The scaling was set to total protein amount with a ploidy of two. The total cellular protein concentration was assumed to be 200 g/L.

##### Network analysis

Reactome pathway network analysis was performed with Cytoscape (version 3.8.0) in combination with the plugins ClueGO (version 2.5.7) and CluePedia (version 1.5.7) (Shannon et al., 2003; Bindea et al., 2009, 2013). Pathway enrichment was determined with a custom reference list containing all mitochondrial proteins identified in the respective dataset. Reactome pathway annotations were retrieved on May 8^th^, 2020.

##### Analysis of MitoBloCK-6 and CCCP data

Data upon MitoBloCK-6 and CCCP treatment was analyzed by differential expression analysis using protein-wise, peptide-based linear mixed effects models (LMM) to obtain log_2_ fold changes and *P values* for treated versus control samples (Goeminne et al., 2015). For each protein, all identified PSMs were used as repeated-measures of the protein. Quantifications were log_2_ transformed and linear regression was fitted with the following formula:

$$y_{i\;}=\;\beta_{0}+\beta X_{i}+u_{i}+\varepsilon_{i}$$

Where *y*_*i*_ denotes expression of peptide *i*, $\beta_{0}$ is the individual protein’s global intercept, *βX*_*i*_ is the linear combination of indicator variables encoding categorical experimental conditions, *u*_*i*_ is the additive random intercept of peptide i with *u*_*i*_ *∼N(0,σ*^*2*^_*u*_*)*, and ε_i_ are residual errors with *ε*_*i*_ *∼N(0,σ*^*2*^_*ε*_*)*.

The model was fitted with the statsmodels 0.12.2 API using Python 3.6 (Seabold and Perktold, 2010). Protein quantifications were restricted to unique peptides and *P values* were adjusted with Benjamini-Hochberg FDR correction.

Visualization of data upon MitoBloCK-6 and CCCP treatment (Figures 3, 4, 5, and S3) was conducted with RStudio software (version 3.6.1) in combination with the tidyverse, ggridges, ggpubr and ggrepel packages (R Core Development Team, 2019; Wickham et al., 2019; Kassambara, 2020; Slowikowski, 2020; Wilke, 2020). Bar graphs were obtained with Microsoft Excel. In Figures 4 and 5, analysis was restricted to proteins for which data was available in all relevant datasets (translation and protein uptake, each in presence of CCCP and CCCP + ISRIB).

## Data Availability

• Mass spectrometry proteomics data have been deposited at the ProteomeXchange Consortium via the PRIDE (Perez-Riverol et al., 2019) partner repository and are publicly available as of the date of publication. Accession numbers are listed in the Key resources table. For detailed experimental description, see Table S6. Original western blot images have been deposited at Mendeley and are publicly available as of the date of publication. The DOI is listed in the Key resources table. Microscopy images shown in Figures have been deposited at Mendeley and are publicly available as of the date of publication. Microscopy data used for quantification will be shared by the lead contact upon request. • All original code for the DynaTMT tool has been deposited at Zenodo and is publicly available as of the date of publication. DOIs are listed in the Key resources table. • Datasets generated during this study and used for Figures 2, 3, 4, 5, and S2–S4 are provided in Tables S1, S2, S3, S4, and S5. Any additional information required to reanalyze the data reported in this paper is available from the lead contact upon request.

## Supplemental information

Document S1. Figures S1–S4

Table S1. Kinetics data of mitochondrial protein uptake, related to Figure 2 Sheet “Slopes” contains normalized labeled protein abundances in both duplicates for each time point and slope, intercept, R^2^ and p value of the regression line. Sheet “Quartiles” contains the mitochondrial proteins assigned to the four quartiles.

Table S2. Reactome pathway network analyses, related to Figures 2, 3, and 5 Sheets contain network analysis summaries obtained with ClueGO of the networks shown in the respective figure panel.

Table S3. Mitochondrial protein uptake upon MitoBloCK-6 treatment, related to Figure 3 Table gives log_2_ of the mean MitoBloCK-6/DMSO ratio, p value, adjusted p value, and -log_10_ of the adjusted p value.

Table S4. Mitochondrial protein uptake upon CCCP treatment, related to Figure 3 Table gives log_2_ of the mean CCCP/DMSO ratio, p value, adjusted p value, and -log_10_ of the adjusted p value.

Table S5. Mitochondrial protein uptake and translation upon CCCP and CCCP+ISRIB treatment, related to Figures 4 and 5 Sheet “Uptake” gives log_2_ of the mean CCCP/DMSO and CCCP+ISRIB/DMSO ratios of protein uptake and the corresponding p value, adjusted p value, and -log_10_ of the adjusted p value. Sheet “Translation” gives log_2_ of the mean CCCP/DMSO and CCCP+ISRIB/DMSO ratios of protein translation and the corresponding p value, adjusted p value, and -log_10_ of the adjusted p value.

Table S6. Experimental design of mass spectrometry proteomics data deposited on PRIDE, related to STAR Methods Table with experimental detail of individual mass spectrometry proteomics data sets deposited on PRIDE, including experiment type, labeling details, and sample order.

Document S2. Article plus supplemental information
