# Supplementary material for: Global mitochondrial protein import proteomics reveal distinct regulation by translation and translocation machinery
Source: Mol Cell. 2022 Jan 20;82(2):435–446.e7. doi: 10.1016/j.molcel.2021.11.004 (PMC8791276; doi:10.1016/j.molcel.2021.11.004)

Molecular Cell, Volume *82*

Supplemental information

Global mitochondrial protein import proteomics reveal distinct regulation by translation and translocation machinery

Jasmin Adriana Schäfer, Süleyman Bozkurt, Jonas Benjamin Michaelis, Kevin Klann, and Christian Münch

Supplemental Information titles and legends

Figure S1. Compartment specific signal boosting of cell-wide and organelle-selective pulsed-SILAC experiments, related to Figure 1

(A) Column graph showing enrichment for summed intensities per compartment.

(B) Column graph of mitochondrial extract content as percentage of summed intensities per compartment.

(C) Density distributions showing enrichment of proteins of different compartments in mitochondrial extract compared to whole cell extract upon proteinase K digestion, derived by TMT-multiplexing (DDA) (n = 3). Dashed lines indicate median values of the distributions.

(D) Density distributions of proteins of different mitochondrial compartments in proteinase K digested mitochondrial isolates compared to non-digested isolates (n = 3). Dashed lines indicate median values of the distributions.

Figure S2. Characterization of protein uptake kinetics across the mitochondrial proteome, related to Figure 2

(A) Plots of mean heavy abundance of TSPO (upper) and NDUFS6 (lower) over time, including least-squared linear and exponential curve fits (red), respectively (n = 2).

(B) Plots of mean light abundance over time. Half-lives were determined by one phase decay nonlinear regression analysis.

(C) Correlations of protein uptake slopes [$\log_2$] with protein length [$\log_2$], GRAVY score and isoelectric point [$\log_2$].

(D) Reactome pathway network showing proteins of Q4 (from Figure 2F), prepared with the Cytoscape plug-in ClueGO. No GO term was significantly enriched after Benjamini-Hochberg correction. Full GO term list is provided in Table S2.

(E) Uptake rates of all identified mitochondrial proteins with highlighted components involved in mitochondrial protein quality control, Krebs cycle, oxidative phosphorylation or of the mitochondrial ribosome. Data points are scattered along the vertical axis to prevent excessive overlapping. PQC, protein quality control; AU, arbitrary units.

Figure S3. Validation of mitochondrial uptake proteomics with alternative assays, related to Figure 3

(A) Western blot of apoptosis markers after 2, 6 and 24 h treatments with CCCP or MitoBloCK-6. 1 mM of staurosporine treatment was used as positive control to induce apoptosis.

(B) Overlap of MTS-EGFP with Mitotracker Red FM in HeLa cells treated with DMSO or CCCP (left). Scale bar: 50 µm. Quantification of cells showing no, low, predominant or complete mitochondrial co-localization of MTS-EGFP with Mitotracker Red FM for 100 EGFP-positive cells per replicate (right, n = 3). Mean + standard deviation. MTR, Mitotracker Red FM.

(C) Volcano plot showing fold changes of mitochondrial protein uptake plotted against the adjusted *P value* for cells treated with MitoBloCK-6 compared to DMSO-treated control cells (n = 3). Data points of described MitoBloCK-6 targets (Dabir et al., 2013; Modjtahedi et al., 2016) were labeled. Data points and labels of targets with significant changes (fold change [$\log_2$] ≤ -0.7 or ≥ 0.7, and adjusted $P \leq 0.05$) are shown in blue. Adjusted *P values* > 10 were set to $P = 10$ for plotting; original adjusted *P values* are given in Table S3.

(D) Comparison of HSPD1 precursor accumulation upon 2 and 6 h of CCCP treatment. p-HSPD1: non-processed precursor HSPD1, m-HSPD1: matrix-localized HSPD1 with removed MTS sequence.

(E) Western blots of mitochondrial proteins showing precursor accumulation upon CCCP treatment. p-, precursor; m-, mature.

(F) Western blots of whole cell lysates showing protein levels of mitochondrial proteins after 6h DMSO or CCCP treatment.

(G) Workflow of the pulsed HaloTag-based mitochondrial protein uptake assay.

(H) Western blots showing newly imported and total HaloTag-labeled proteins after 6 h CCCP treatment (left). CCCP/DMSO ratios were compared to ratios obtained with $mePROD^{mt}$ (right). Here, a protein-based quantification of the log_2 fold change was used, as our LMM quantification does not generate replicate specific fold changes.

(I) Correlation of CCCP-induced fold changes of mitochondrial protein uptake and steady-state uptake rates.

Figure S4. Stress shapes mitochondrial protein uptake via translation and import regulation, related to Figure 4

(A) Correlations of fold changes of mitochondrial protein uptake upon CCCP + ISRIB treatment compared to DMSO [log_2] with protein length [log_2], GRAVY score, isoelectric point [log_2] and protein copy number.

(B) Density plot showing fold changes of mitochondrial proteins in the mitochondrial fraction and whole cell lysate upon MG-132 + CCCP treatment compared to CCCP-treated cells (n = 3). Dashed lines indicate median values of the distributions.

Figure S1: Compartment specific signal boosting of cell-wide and organelle-selective pulsed-SILAC experiments, related to Figure 1

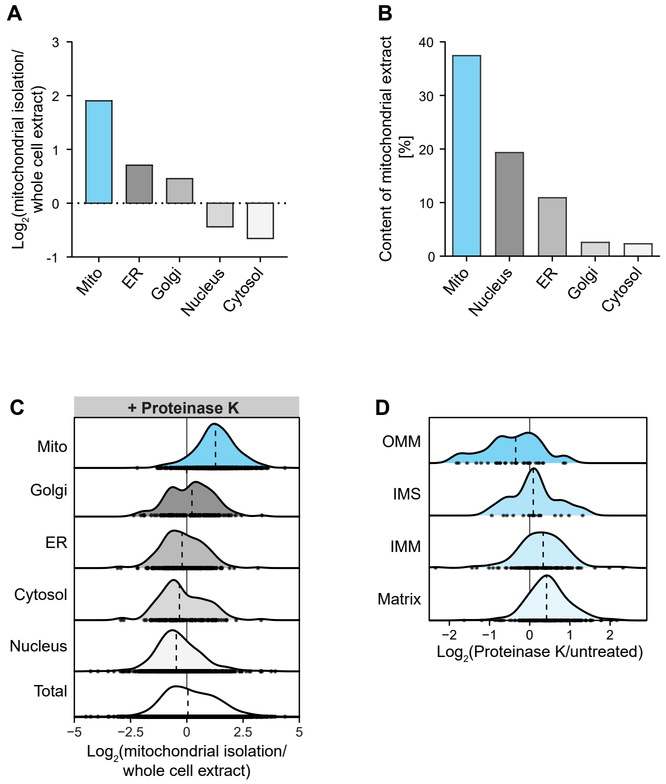

Figure S2: Characterization of protein uptake kinetics across the mitochondrial proteome, related to Figure 2

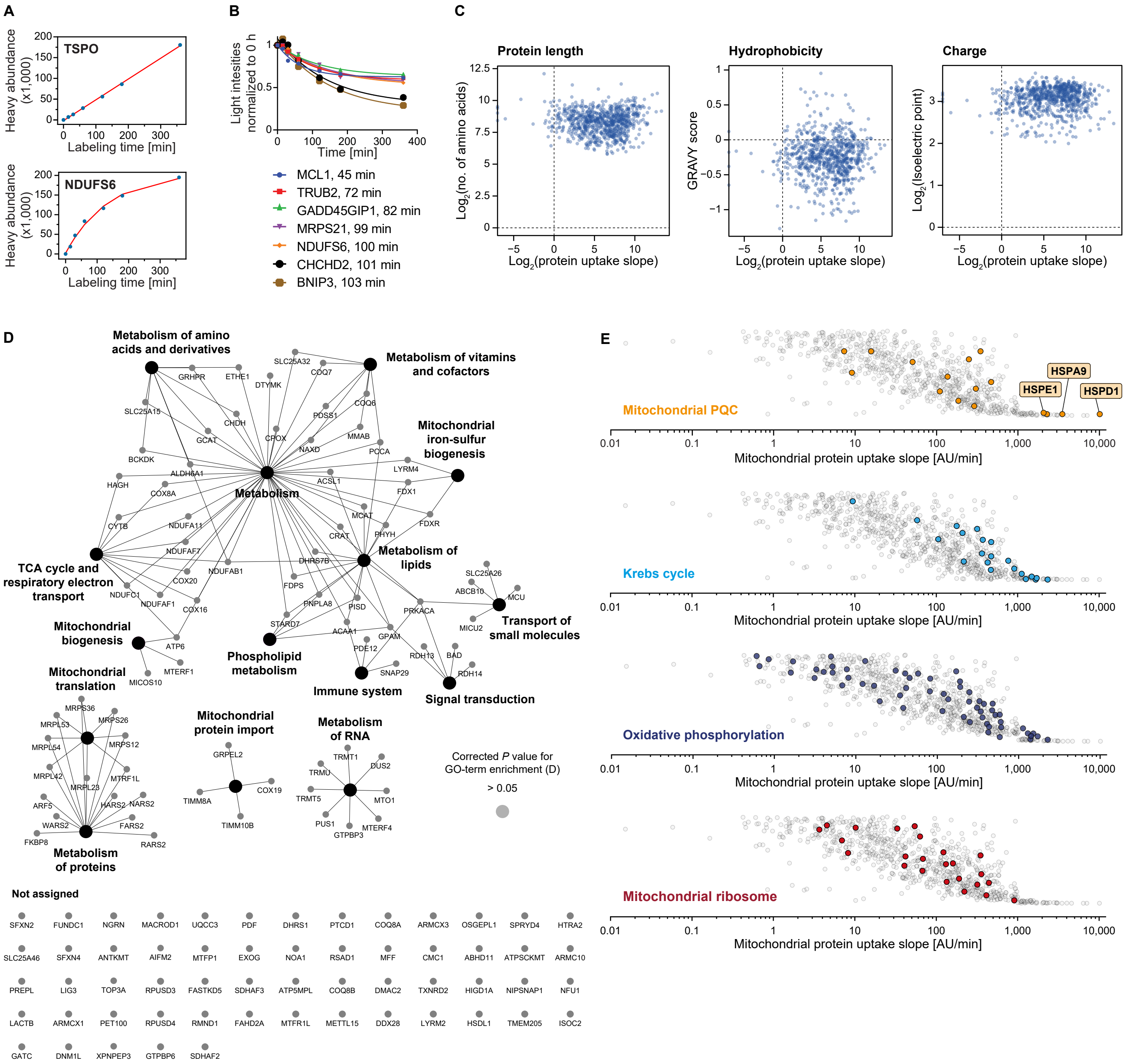

Figure S3: Validation of mitochondrial uptake proteomics with alternative assays, related to Figure 3

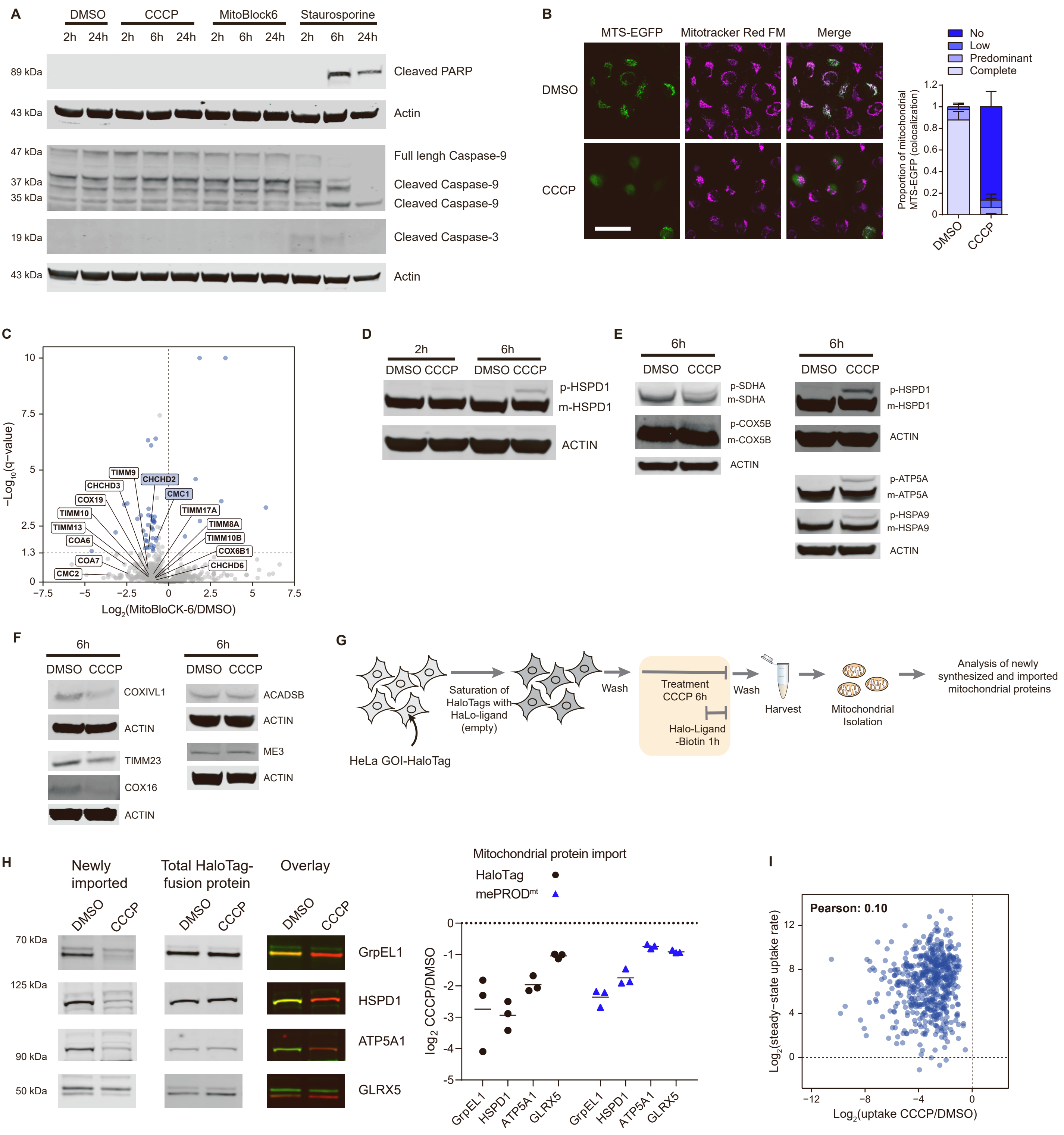

Figure S4: Stress shapes mitochondrial protein uptake via translation and import regulation, related to Figure 4

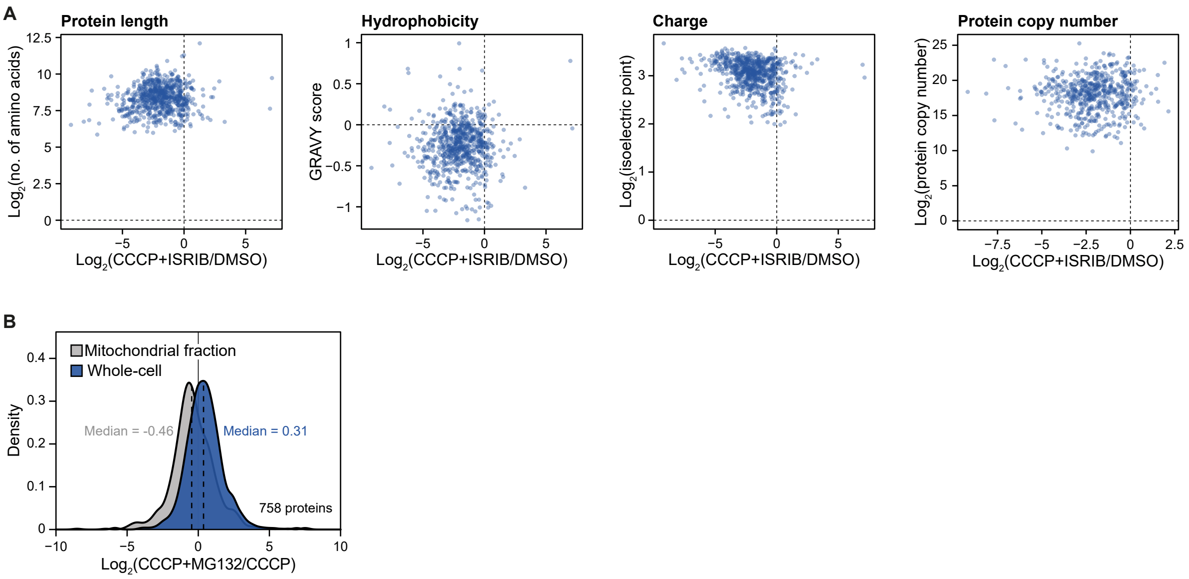

Supplement: Document S1. Figures S1–S4 [file mmc1.pdf]
